# A Rare Primary Neuroendocrine Tumor (Typical Carcinoid) of the Sublingual Gland

**DOI:** 10.1155/2016/7462690

**Published:** 2016-10-20

**Authors:** Kenji Yamagata, Kousuke Ohki, Fumihiko Uchida, Naomi Kanno, Shogo Hasegawa, Toru Yanagawa, Hiroki Bukawa

**Affiliations:** ^1^Department of Oral and Maxillofacial Surgery, Institute of Clinical Medicine, Faculty of Medicine, University of Tsukuba, Tsukuba, Japan; ^2^Department of Oral and Maxillofacial Surgery, Ishioka Daiichi Hospital, Ishioka, Japan

## Abstract

A typical carcinoid is extremely rare in the oral cavity. We here present a case of a typical carcinoid arising in the sublingual gland of a 62-year-old woman. The tumor was removed by primary excision with 10 mm surgical margins and submandibular dissection. Examination of the tumor showed medium-sized tumor cells that were positive for CD56 and chromogranin A, with no necrosis, and with a mitotic count less than 1/10 HPF. A pathological diagnosis of typical carcinoid was made from both morphological and immunological examinations. One year after excision surgery, there was no tumor recurrence or neck metastasis.

## 1. Introduction

Neuroendocrine tumors (NETs) are heterogeneous, ranging from benign to highly malignant. In the larynx, the most frequent NETs are atypical carcinoids (53.7%), followed by neuroendocrine-type small cell carcinomas (27.6%), paragangliomas (12.1%), and typical carcinoids (6.6%) [1]. The typical carcinoid, sometimes simply referred to as a carcinoid, is a well-differentiated neuroendocrine carcinoma (Grade I) with sparse to absent nucleoli and mitoses [less than 2/10 high-power fields (HPF)] and no necrosis or pleomorphism [2].

Most carcinoids occurring in the head and neck area are found in the larynx, followed by the middle ear [3]. Yang et al. reported the first case of a typical carcinoid in the oral cavity in 2011 [4]. To our knowledge, only 2 cases of typical carcinoids arising in the oral cavity have been reported [4, 5]. We here present an extremely rare case of a typical carcinoid arising in the sublingual gland.

## 2. Case Report

**Figure 1:**
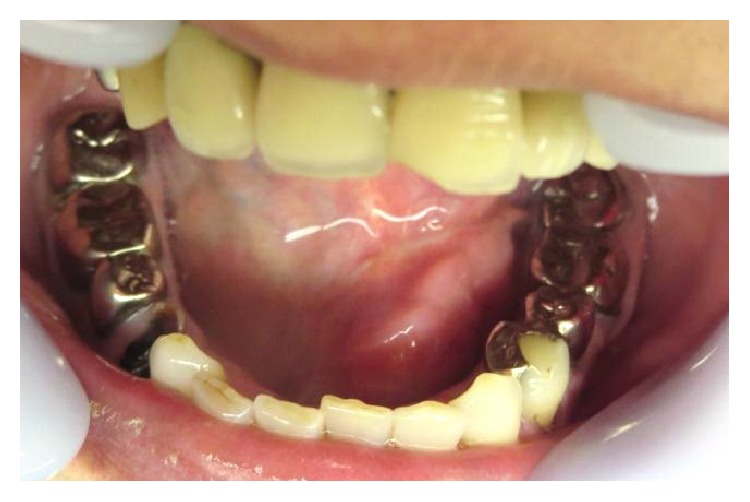
Intraoral examination. Examination of the oral cavity showed a 28 × 13 mm hard elastic mass in the left floor of the mouth. The mucosal surface was normal, with no ulceration.

A 62-year-old woman came to the Department of Oral and Maxillofacial Surgery, University of Tsukuba Hospital, with a clinical diagnosis of a sublingual gland tumor. She had noticed swelling in the sublingual gland, with no pain, for 9 months. She had a history of hypertension, cerebral infarction, and hyperlipidemia. Her face was symmetrical and there was no trismus. The regional lymph nodes were normal, and the submandibular gland was hard and swollen. Examination of the oral cavity showed a hard, elastic mass measuring 28 × 13 mm on the left side of the floor of her mouth. The mucosal surface was normal, with no ulceration (Figure 1). There was no observable saliva flow from the opening of Wharton's duct.

**Figure 2:**
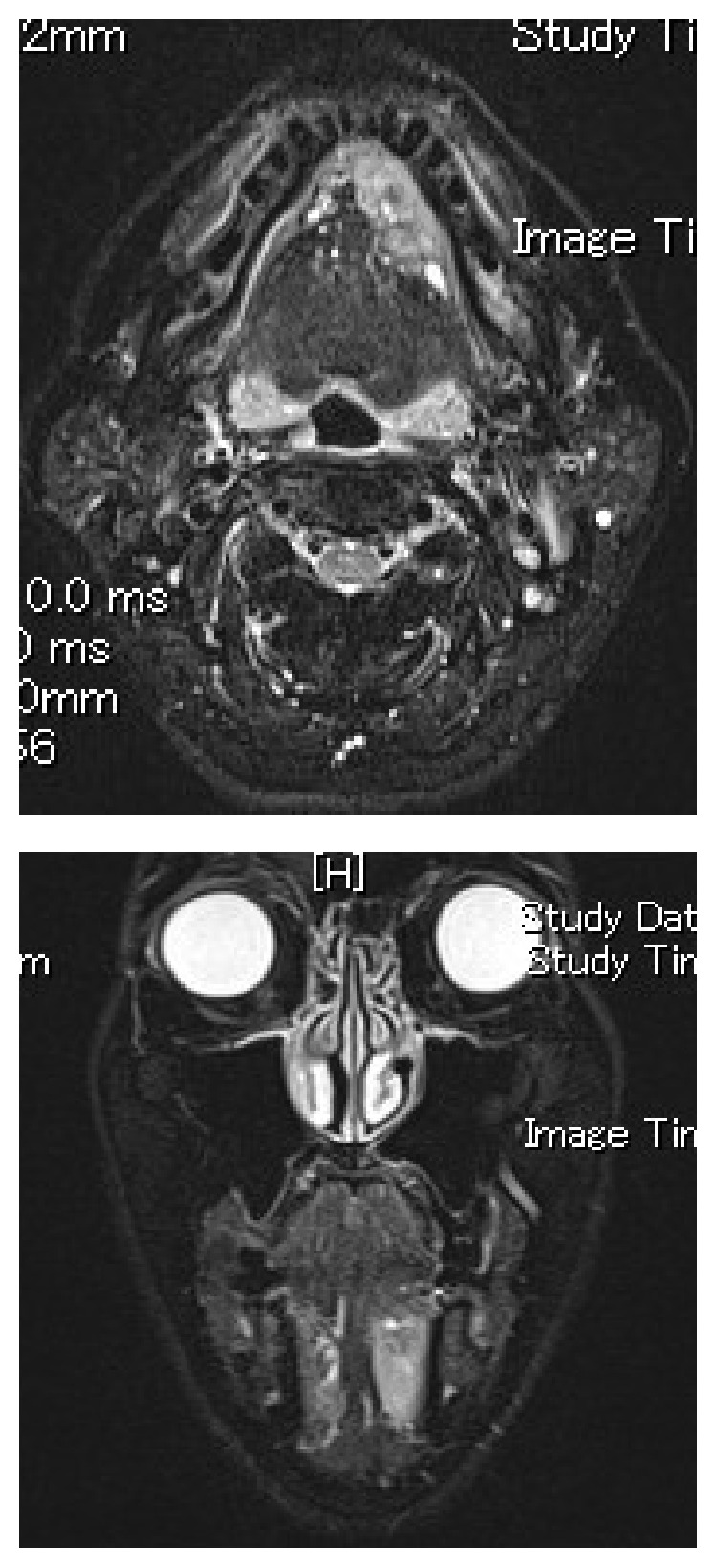
MR images (STIR) showed a well-defined tumor of the sublingual gland with a high-signal mass measuring 28 × 25 × 12 mm.

**Figure 3:**
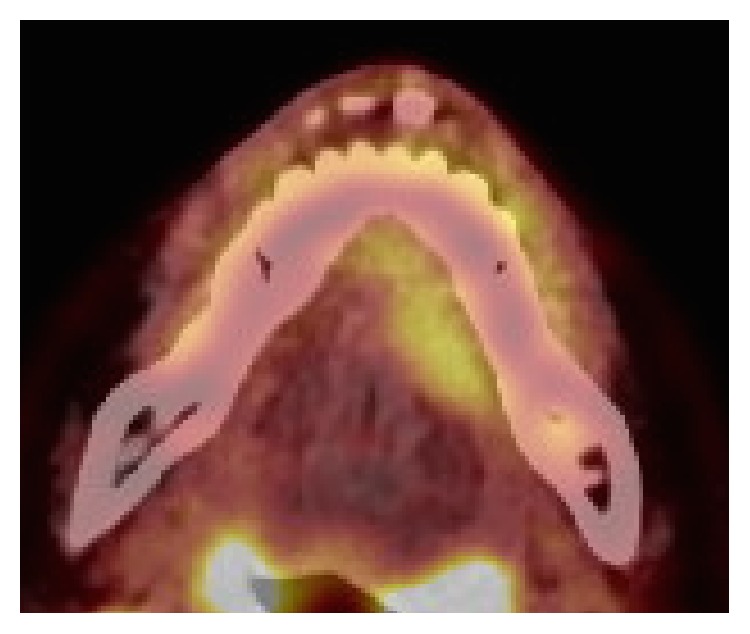
FDG-PET. The sublingual mass measured 28 × 13 mm and had a max SUV of 10.4.

Magnetic resonance (MR) images [short TI inversion recovery (STIR)] showed a well-defined tumor of the sublingual gland with a high-signal mass measuring 28 × 25 × 12 mm (Figure 2). Fluorodeoxyglucose positron emission topography (FDG-PET) depicted a mass measuring 28 × 13 mm with a max standard uptake value (SUV) of 10.4 (Figure 3) and did not show any neck metastasis or distant metastasis.

The carcinoid was removed by primary excision performed extraorally under general anesthesia, with 10 mm surgical margins and submandibular dissection with the level I lymph node and submandibular gland. The intraoral resected defect was partially sutured and covered with polyglycolic acid (PGA) sheets and fibrin glue. The postoperative course was uneventful. One year later, there was no tumor recurrence or neck metastasis.

**Figure 4:**
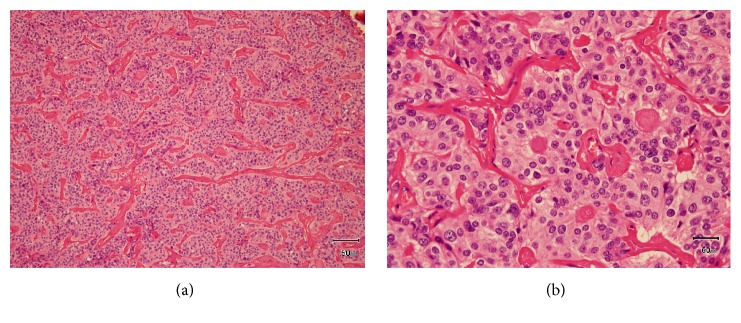
HE staining with low power (a) and high power (b). The round cells were arranged as cords, solids, trabeculae, and nests with a highly vascularized, hyalinized stroma. There was no necrosis, and the mitotic count was <1/10 HPF.

**Figure 5:**
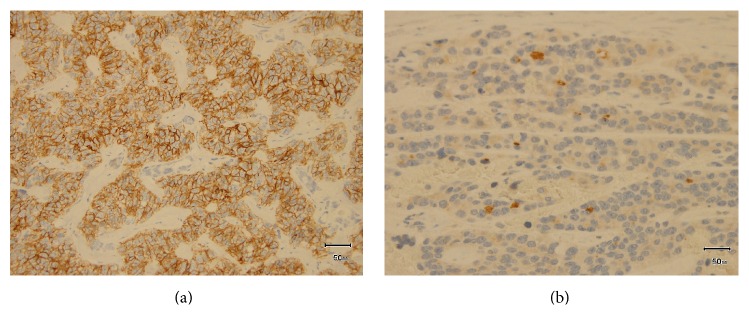
Immunohistochemical staining. The staining was positive for CD56 (a) and slightly positive for chromogranin A (b).

Histological examination revealed that the resected tumor, which was 26 × 24 × 12 mm, was whitish in color and solid in consistency. The round cells were solid or arranged in cords, trabeculae, or nests, with hyalinized stroma with high vascularization. The tumor cells had eosinophilic cytoplasm. The nuclei were round and varied in size, and the rough chromatin and mitotic count was less than 1/10 HPF. There was no necrosis (Figure 4). The tumor had partly infiltrated the sublingual gland and had no clear boundary. Immunohistochemical staining showed that the tumor was CD56 (+), chromogranin A (+), and synaptophysin (−) (Figure 5). The Ki-67 index was 2.8%. A pathological diagnosis of typical carcinoid of the sublingual gland was made based on the morphological and immunological exam. The surgical margin was free, and no lymph node metastasis was found.

## 3. Discussion

Carcinoid tumors are the most common of the NETs. They are usually found in the gastrointestinal tract (55%) or the bronchopulmonary tract (10%) [6]. In 2005, WHO classified NETs of the larynx into 4 types: (1) typical carcinoid, (2) atypical carcinoid, (3) small cell carcinoma, neuroendocrine type, and (4) combined small cell carcinoma, neuroendocrine type, with non-small cell carcinoma. The typical carcinoid tumor is defined as “a tumor with a neuroendocrine/carcinoid morphology, mitotic rate of less than 2/10 HPF, and absence of necrosis” [2]. Typical carcinoids, which are the least common NETs in the head and neck area, occur most commonly in the supraglottic larynx, with rare examples in the parotid gland and sinonasal tract. Because typical carcinoids are relatively rare, it is still difficult to describe their clinical behavior or prognosis [7]. The reported age at presentation ranges from 23 to 71 years, and most cases report no tumor recurrence at follow-up (18 to 124 months after treatment) [7].

Typical carcinoids are extremely rare in the head and neck region [8]. Only 14 NETs originating in the oral region have been reported in the English-language literature [9, 10]; of these, atypical carcinoid tumors were the most common (6 cases), followed by neuroendocrine- type small cell carcinoma (4), typical carcinoids (2), and other (2). In the 2 reported cases of typical carcinoid of the oral cavity [4, 5], the primary sites were the mandible and retromolar regions, and both cases were treated by excision under general anesthesia. In both cases, the disease did not recur during the follow-up period (2 years in one case and 11 months in the other) (Table 1). Although there is one report of a nontypical carcinoid in the floor of the mouth [11], this is the first report of a typical carcinoid arising in the floor of mouth.

Our case was diagnosed as a typical carcinoid based on our findings and the algorithm described by Mahomed [12] for diagnosing oral NETs. The typical carcinoid is diagnosed based on tumor cells that are medium or large in size, positive for epithelial and neuroendocrine markers, free of necrosis, and having a mitotic rate less than 2/10 HPF. Typical carcinoid tumors are composed of round or spindle-shaped cells, or both, with histologic and immunohistologic evidence of neuroendocrine differentiation.

According to the 2005 WHO classification of NETs of the larynx, atypical carcinoid tumors exhibit more mitotic and more atypical cells than do the typical carcinoids [2]. Typical carcinoid tumors are composed of round or spindle-shaped cells that grow in trabeculae, gland-shaped structures and/or rosettes, small nests, or large sheets. Mitosis is sparse to absent. The stoma is highly vascularized and is often focally fibrotic or hyalinized [2]. Immunohistochemically, NETs are positively stained by one or more neuroendocrine markers [12], and chromogranin A, synaptophysin, CD57, CD56, neuron specific enolase, and neurofilaments are important for evaluating neuroendocrine neoplasms. In the present case, the medium-size tumor cells were positive for CD56 and chromogranin A, had no necrosis, and had a mitotic count less than 1/10 HPF.

Previously reported NETs have been treated by tumor resection. Radiation and chemotherapy are ineffective against typical carcinoids, making surgery the treatment of choice. NETs should be resected as conservatively as possible while still removing the tumor completely. A neck dissection is not warranted. In the present case, the sublingual tumor extended deeply toward the border of the mandible. The excision was performed from the submandible extraorally with submandibular dissection. Moreover, the patient was in her sixth decade and was being treated surgically. The reported rate of metastases for a carcinoid of the larynx is 33%, and the 5-year survival rate is 48% [1, 2]. In all 3 reported cases of oral typical carcinoid, including the present case, the carcinoid was treated by excision and did not recur during the follow-up period (1-2 years).

## Figures and Tables

**Table 1:** Reported cases of oral typical carcinoid.

Reference	Age/gender	Site	Size (mm)	Treatment	Prognosis (M)
Coleman et al. [5]	46/F	Mandible	40 × 38 × 50	Excision	NED (24)
Yang et al. [4]	46/F	Retromolar region	15 × 20	Excision	NED (11)
Present case	62/F	Floor of mouth	28 × 25 × 12	Excision	NED (12)

NED: no evidence of disease.
